# COVID-19 Outbreaks and Control Measures Are Associated With Depression Risk Among Older Adults

**DOI:** 10.1093/geroni/igab046.1470

**Published:** 2021-12-17

**Authors:** Man-Man Peng, Tianyin Liu, Walker Siu Hong Au, Terry Y S Lum, Gloria H Y Wong

**Affiliations:** 1 Beijing Normal University at Zhuhai, China, Zhuhai, Guangdong, China (People's Republic); 2 The University of Hong Kong, Hong Kong, Hong Kong

## Abstract

Local COVID-19 outbreaks and infection control measures may affect mental health in older persons. This study aims to investigate the effects of COVID-19 outbreaks and control measures on depression risk in community-dwelling older adults in Hong Kong. With rolling cross-sectional design, telephone screenings for depressive risk were conducted among 8163 older people using Patient Health Questionnaire-2 (PHQ-2) from February to September 2020. COVID-19 outbreaks across thirty weeks were measured using real-time effective reproductive number (Rt), infected new cases, and change of infected new cases by week. Infection control measures were assessed using four policy indices, including government response, government stringency, containment and health, and economic support. Linear regressions were used to test the associations of depression risk with COVID-19 outbreaks and control measures. We found that being female and higher Rt were associated with higher depression risk in the overall sample. In older adults without pre-existing mental health issues, higher depression risk were related to older age (t=-1.974, 95%CI[-0.006, 0.000], p<0.05), a higher level of government stringency (t=2.954, 95%CI[0.007, 0.033], p<0.01), and less stringent containment and health-related policy (t=-2.599, 95%CI[-0.041, -0.006], p<0.01). In older adults with pre-existing mental health issues, greater changes in newly infected cases were related to higher depression risk (t=2.813, 95%CI[0.002, 0.010], p<0.01). In conclusion, the effects of COVID-19 infection risk and control measures on depression risk differ among older Chinese by pre-existing mental health issues. Future public health communication could build on resilience to balance awareness of infection risks and mental health risks in older persons.

